# Hyperfractionated-Accelerated Reirradiation with Proton Therapy for Radiation-Associated Breast Angiosarcoma

**DOI:** 10.14338/IJPT-21-00031.1

**Published:** 2022-01-18

**Authors:** Wen Shen Looi, Julie A. Bradley, Xiaoying Liang, Christiana M. Shaw, Mark Leyngold, Raymond B. Mailhot Vega, Eric D. Brooks, Michael S. Rutenberg, Lisa R. Spiguel, Fantine Giap, Nancy P. Mendenhall

**Affiliations:** 1 University of Florida Health Proton Therapy Institute, Jacksonville, FL, USA; 2 Department of Radiation Oncology, University of Florida College of Medicine, Jacksonville, FL, USA; 3 Department of Radiation Oncology, Mayo Clinic, Jacksonville, FL, USA; 4 Department of Surgery, University of Florida College of Medicine, Gainesville, FL, USA

**Keywords:** angiosarcoma, breast cancer, second malignancies, hyperfractionated radiation, cancer outcomes

## Abstract

**Purpose:**

Radiation-associated angiosarcoma (RAAS) is a rare complication among patients treated with radiation therapy for breast cancer. Hyperfractionated-accelerated reirradiation (HART) improves local control after surgery. Proton therapy may further improve the therapeutic ratio by mitigating potential toxicity.

**Materials and Methods:**

Six patients enrolled in a prospective registry with localized RAAS received HART with proton therapy between 2015 and 2021. HART was delivered twice or thrice daily in fraction sizes of 1.5 or 1.0 Gy, respectively. All patients received 45 Gy to a large elective volume followed by boosts to a median dose of 65 (range, 60-75) Gy. Toxicity was recorded prospectively by using the Common Terminology Criteria for Adverse Events, version 4.0.

**Results:**

The median follow-up duration was 1.5 (range, 0.25-2.9) years. The median age at RAAS diagnosis was 73 (range, 60-83) years with a median latency of 8.9 (range, 5-14) years between radiation therapy completion and RAAS diagnosis. The median mean heart dose was 2.2 (range, 0.1-4.96) Gy. HART was delivered postoperatively (n = 1), preoperatively (n = 3), preoperatively for local recurrence after initial management with mastectomy (n = 1), and as definitive treatment (n = 1). All patients had local control of disease throughout follow-up. Three of 4 patients treated preoperatively had a pathologic complete response. The patient treated definitively had a complete metabolic response on her posttreatment PET/CT (positron emission tomography–computed tomography) scan. Two patients developed distant metastatic disease despite local control and died of their disease. Acute grade 3 toxicity occurred in 3 patients: 2 patients undergoing preoperative HART experienced wound dehiscence and 1 postoperatively developed grade 3 wound infection, which resolved.

**Conclusion:**

HART with proton therapy appears effective for local control of RAAS with a high rate of pathologic complete response and no local recurrences to date. However, vigilant surveillance for distant metastasis should occur. Toxicity is comparable to that in photon/electron series. Proton therapy for RAAS may maximize normal tissue sparing in this large-volume reirradiation setting.

## Introduction

Breast-conservation therapy (BCT) using a combination of lumpectomy and adjuvant radiation therapy (RT) has reduced the need for mastectomy in women with early-stage breast cancer. Rarely do patients develop toxicities after BCT that can result in significant morbidity and present treatment challenges. Radiation-associated angiosarcoma (RAAS), however, is a rare secondary malignancy with an incidence of 0.03% to 0.3% [1, 2] and a median latency period of 5 to 9 years after RT [1, 3–16]. RAAS is an aggressive tumor with a propensity towards local recurrence and tends to have a poor prognosis [5, 10, 12, 16–18]. When compared to primary breast angiosarcoma, local control is poor, although distant metastases are less frequent [12]; it has a shorter latency period [11] and often afflicts older women, with a median age around 70 years [1, 5–10, 12–16, 19, 20]. These women are more likely to have comorbid medical conditions that complicate management. Moreover, owing to its rarity, there is a paucity of data to guide management beyond single-institution retrospective series.

Surgical excision with wide margins is the mainstay of treatment for localized RAAS. Mastectomy is the most common surgery; unfortunately, local recurrence is common even with complete resection [6, 21, 22], possibly owing to diffuse infiltration or multifocality [23]. After surgery alone, local recurrence rates can reach 92% [7, 12, 14, 15, 18, 24–26]. These recurrences can be rapid and often occur near or at the surgical incision [3, 22, 25, 27, 28]. RAAS is a radiosensitive tumor, and adjuvant RT has been recommended to improve outcomes [13, 21, 24–26, 29, 30]. Despite its purported benefits, adoption of routine RT for RAAS has been limited over concerns for high cumulative doses to normal tissues.

Hyperfractionated-accelerated reirradiation (HART) is an approach that allows for safe radiation delivery and has demonstrated favorable long-term outcomes with a 10-year cause-specific survival rate of 71% [3]. HART using proton therapy is particularly advantageous in patients with left-sided disease and permits significant cardiac sparing, mitigating the known cardiovascular risk related to RT [31]. Additionally, protons can reduce the lung dose in this patient population at risk for radiation fibrosis and radiation pneumonitis. Herein, we analyze and describe our experience using proton therapy to deliver HART for RAAS.

## Materials and Methods

### Patient Population

Under an institutional review board–approved registry study, we identified patients treated with proton therapy for RAAS between 2015 and 2021. Eligible patients had received adjuvant RT after lumpectomy as part of BCT. Histologic confirmation of diagnosis was required, and all patients consented to have their data collected and analyzed. We excluded patients with primary breast angiosarcoma.

### Staging and Treatment

For all patients, disease was staged with positron emission tomography (PET)/computed tomography (CT) before RT with or without magnetic resonance imaging of the breast and mammography. Treatment intent was curative in all patients and incorporated HART, as previously described [3, 4, 27]. Patients referred after surgery received postoperative HART; however, when possible, we favored preoperative HART as it (1) allows for maximal excision of twice-irradiated tissue; (2) allows for downsizing of the tumor that may facilitate an R0 resection, which is associated with improved outcomes [19]; and (3) reduces the risk of rapid and early recurrence during convalescence after mastectomy [3, 18, 22, 25, 27, 28].

Whether HART was delivered preoperatively or postoperatively, the use of autologous tissue reconstruction techniques following oncologic resection was encouraged to facilitate wound healing [3]. Patients underwent a mastectomy or radical chest wall resection. HART was defined as definitive if gross disease was present during RT without plans for surgery. Patients did not undergo surgery if they were poor surgical candidates or declined surgery. Following preoperative radiation, surgery was performed between 4 and 8 weeks after radiation. The surgeon removed the initial extent of gross disease with a minimum margin of 5 cm.

**Figure 1.**
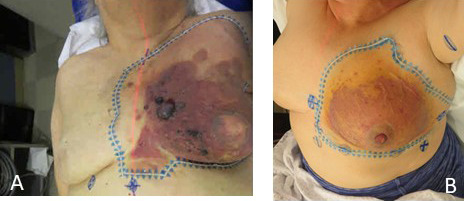
Delineation of gross disease with radio-opaque wire during computed tomography simulation for patient 2 (A) and patient 5 (B). The skin must be closely examined for erythema and edema.

For radiation, all patients were positioned supine on a breast board with arms overhead, and clinically evident disease was marked with radio-opaque wires (**Figure 1**). A 4-dimensional CT simulation was used to assess the impact of respiratory motion on dose distribution and contours were drawn on the average phase. Clinical findings informed the gross tumor volume (GTV) supplemented by radiographic findings, and all skin thickening was included in the GTV. Tattoos (typically 4-6) were placed at the edges of the gross disease to provide a reference for the surgeon as to the initial extent of disease. We used an elective dose of 45 Gy to treat tissues at risk for a low-disease burden; a margin of 5 to 10 cm was applied to the GTV and confined to anatomic barriers of spread. Boosts were delivered sequentially—the patients receiving preoperative HART received further boosts to a total dose of 60 to 70 Gy to the gross disease with a 2-cm margin; patients with gross disease without planned surgical excision received definitive total doses of 72 to 75 Gy to gross disease.

**Figure 2.**
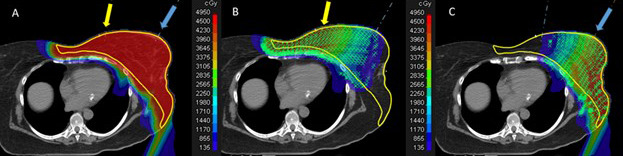
(A) PBS proton-dose colorwash diagram of the first phase of HART, delivering 45 GyRBE. The clinical target volume is outlined in yellow. The yellow and blue arrows indicate the direction of the en face beams with gantry angles of 10° and 30°, respectively. (B) The same dose colorwash with green crosses and green circles indicating PBS spot placement from the medial beam. The spot weighting is proportional to the circle size. (C) The same dose colorwash with green crosses and green circles indicating PBS spot placement from the lateral beam. The spot weighting is proportional to the circle size. A smooth dose gradient was planned across the overlap region of the 2 beams; a distinct matchline is not required for PBS. Abbreviations: HART, hyperfractionated-accelerated reirradiation; PBS, pencil-beam scanning.

Proton therapy was delivered with either pencil-beam scanning (PBS) or double-scatter proton modalities. Generally, 2 en face beams provided target coverage, a technique previously described for breast carcinoma proton therapy [32]. Individualization of treatment fields was required as target volumes were often more extensive than standard breast target volumes. Proton therapy delivered with PBS obviates the need for matchlines and may mitigate some of the dosimetric uncertainty (**Figure 2**). As skin was part of the target, there was no intentional modulation of skin dose during treatment planning. A bolus was unnecessary as sufficient skin dose is achieved with proton therapy for superficial targets. Combined-modality treatment involving photons and/or electrons was used in some cases to accelerate the RT start and/or to achieve the required 4-hour interfraction period.

### Disease Control and Toxicity

Acute and late toxicities were graded prospectively per the National Cancer Institute's Common Terminology Criteria for Adverse Events, version 4.0 (US National Cancer Institute, Bethesda, Maryland). Patients were monitored weekly for acute toxicity while on treatment. Patients were seen 2 to 3 times/wk during RT (based on twice-daily [BID] versus thrice-daily [TID] fractionation) and 1 to 3 times/wk for at least 2 weeks after completing RT and discharged to regular follow-up once toxicity improved to grade 2 or lower. A weekly basic metabolic panel was obtained to assess electrolytes and kidney function in the setting of extensive dermatitis given the large field sizes.

## Results

### Patient and Tumor Characteristics

Between 2015 and 2021, six patients with RAAS underwent HART. All had undergone BCT involving lumpectomy, axillary surgery, and adjuvant whole-breast RT. The median age at RAAS diagnosis was 73 (range, 60-83) years with a median latency of 8.9 (range, 5-14) years between completion of RT and RAAS diagnosis. All 6 patients had a baseline Zubrod performance status score of 0 or 1 at the time of HART. The median tumor size was 16 (range, 10.8-17) cm. The median follow-up duration from initial biopsy was 1.7 (range, 0.6-2.9) years and no patients were lost to follow-up.

**Figure 3.**
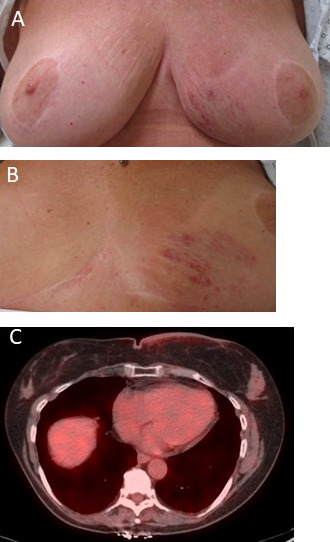
Photographs of a patient with radiation-associated angiosarcoma of the left lower inner breast showing violaceous skin changes, erythema, and superficial edema in the (A) seated and (B) supine positions. (C) An axial image of the staging positron emission tomography/computed tomography study of the same patient demonstrating extensive skin thickening with moderate fluorodeoxyglucose tracer uptake of the medial left breast.

All patients had left-sided breast RAAS. The median duration from the onset of symptoms to RAAS diagnosis was 2.5 (range, 1-6) months. On clinical examination, all patients had skin changes in coloration or texture described as erythematous, violaceous, thickened, and/or ecchymotic (**Figure 3A** and **3B**). No patients had extremity lymphedema identified before RT. One patient had associated benign dermal sclerosis/morphea, which was histologically confirmed 11 months before the diagnosis of RAAS without evidence of angiosarcoma at that time.

Four of the 6 patients had screening mammograms within 12 months of diagnosis, which were negative for malignant changes. The patient with a history of dermal sclerosis had evidence of skin thickening on mammogram a year prior. The next mammogram demonstrated worsened skin thickening, which prompted further workup that led to the diagnosis of RAAS (patient 5). PET/CT demonstrated fluorodeoxyglucose avidity in areas of skin thickening in all patients (**Figure 3C**). Two patients underwent magnetic resonance imaging of the breast with primary findings of skin thickening and enhancement. No patient had clinical or radiologic evidence of nodal or metastatic RAAS.

All patients had biopsy-proven RAAS. CD31 immunohistochemical staining was positive in all cases; CD34 and MYC amplification [33–35] were positive in all cases in which they were evaluated (4 and 3 cases, respectively). Tumor grade was reported for 3 patients and was high grade.

### Treatment

All patients received 45 Gy to a large elective volume followed by boosts to a median dose of 65 (range, 60-75) Gy. The **Table** includes further treatment details for all 6 patients and **Supplemental Figures S1** through **S6** depict each patient's clinical presentation. The 4 patients who received HART in the preoperative setting received total doses from 60 to 70 Gy (patients 1, 3, 5 and 6). The median interval from diagnosis to preoperative HART was 7 (range, 4-14) weeks, while the median duration between completion of HART and surgery was 49 (range, 30-52) days. Three of the 4 patients treated with preoperative HART underwent reconstruction with autologous tissue transfer techniques (ie, flap reconstruction; see **Supplemental Figure S7**). One of these patients (patient 6) had initial treatment with mastectomy (tumor, 10.8 cm) with negative margins (closest was 0.3 cm inferior), followed by rapid local recurrence; she then received preoperative proton therapy to 60 Gy before chest wall resection with reconstruction.

**Table.** Patient treatment details. See Supplemental Figures S1 through S6 for images of clinical presentation.

**Pt**	**Breast cancer management**	**Age at angiosarcoma diagnosis, y**	**Latency period, y**	**Symptom duration, mo^a^**	**Angiosarcoma management**	**HART details**	**Treatment response**	**Mean heart dose, Gy**	**Toxicity**	**Time to event, mo^b^**	**Follow-up duration, mo^b^**	**Status**
1	BCT + SLNB + RT + HT	76	10.8	3	Pre-op HART + simple MT without reconstruction	60 GyRBE in 1.5-GyRBE fractions BID; PBS only	pPR, 0.7 cm residual	0.1	Grade 2 wound dehiscence after MT	N/A	15	ANED
2	BCT + SLNB + RT + HT	83	7.0	1	Definitive HART	75 Gy in 1-Gy fractions TID to main lesion (14 Gy IMRT, 61 GyRBE PBS); 68 Gy to contralateral disease that developed during treatment (21 Gy electron, 47 GyRBE PBS)	PET/CT complete response	4.96	Acute grade 3 dermatitis and fatigue; grade 2 bilateral pleural effusions 5 mo with subsequent diagnosis of CHF, managed medically; grade 2 superficial soft tissue fibrosis	N/A	16	ANED
3	BCT + ALND + RT + HT + CT	67	12.0	6	Pre-op HART + MT with radical resection of chest wall + pedicled rectus myocutaneous flap reconstruction	60 Gy in 1-Gy fractions TID (25 Gy 3D photons matched to electron field; 35 GyRBE DS)	pCR	3	Grade 3 wound infection after MT	N/A	34	ANED
4	BCT+ SLNB + RT + HT	78	14.0	2	MT followed by local chest wall recurrence treated with definitive HART	72 Gy in 1-Gy fractions TID (45 Gy matched electron fields; 27 GyRBE PBS)	Complete clinical response	Electrons planned clinically (no DVH); 0.1 Gy from PBS	Grade 3 acute dermatitis, persisted as chronic grade 2 wound complication in the setting of chemotherapy for metastatic progression	5 (lung metastases)	19	DWD
5	BCT + SLNB + RT + HT	60	6.0	5	Pre-op HART + MT with free right latissimus flap and skin graft reconstruction	70 GyRBE at 1 GyRBE per fraction TID; DS	pCR	2.7	Acute grade 3 dermatitis; grade 2 superficial soft tissue fibrosis	19 (bone metastases)	21	DWD
6	BCT + unknown axillary surgery + RT + HT	69	5.0	1	MT followed by local chest wall recurrence treated with pre-op HART + radical chest wall resection with pedicled latissimus and skin graft reconstruction	60 GyRBE at 1 GyRBE per fraction TID; PBS	pCR	0.7	Acute grade 3 fatigue; grade 2 wound dehiscence after radical chest wall excision with reconstruction	N/A	7	ANED

**Abbreviations:** Pt, patient; HART, hyperfractionated-accelerated reirradiation; BCT, breast conservation therapy; SLNB, sentinel lymph node biopsy; RT, radiation therapy; HT, hormonal therapy; Pre-op, preoperative; MT, mastectomy; BID, twice daily; PBS, pencil-beam scanning protons; pPR, pathologic partial response; N/A, not available; ANED, live with no evidence of disease; TID, three times daily; IMRT, intensity-modulated radiation therapy; PET/CT, positron emission tomography/computed tomography ; CHF, congestive heart failure; ALND, axillary lymph node dissection; CT, computed tomography; DS, double-scatter protons; pCR, pathologic complete response; DVH, dose-volume histogram; DWD, died with disease.

a Before diagnosis of radiation-associated angiosarcoma.

b Calculated from date of angiosarcoma diagnosis.

The patient who received postoperative HART for salvage therapy had undergone mastectomy without autologous tissue transfer reconstruction as initial treatment for RAAS. Surgical margins were negative by 1.5 cm. During her RT consultation 9 weeks after mastectomy, she was observed to have clinical findings concerning for early recurrence—erythema and skin thickening/induration adjacent to the mastectomy incision. She did not have histologic confirmation of recurrence before starting HART. She started postoperative RT 10 weeks after mastectomy and received a total dose of 72 Gy.

One patient declined a mastectomy and underwent definitive HART alone 7 weeks after diagnosis (patient 2). She received a total of 75 Gy. Despite >5-cm margins on gross disease, the patient's disease progressed in the contralateral medial breast adjacent to the target volume and required adaptation of treatment volumes while on treatment. The new gross disease was boosted by an electron field designed by clinical setup to a total dose of 68 Gy. The intended total dose to this new area was 75 Gy, but treatment was stopped early owing to patient fatigue.

Five patients received HART in 1-Gy fractions TID, while 1 patient received 1.5 Gy BID owing to patient refusal of TID fractionation. Three patients received proton therapy alone, while mixed-modality treatment with photons and/or electrons was used in 3 patients (**Table**). No patient received planned systemic therapy. All patients had visible regression of the tumor during RT.

**Figure 4.**
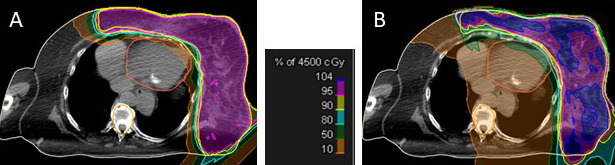
Dose colorwash depicting the initial phase to 45 Gy for (A) a PBS treatment plan (mean heart dose, 0.9 GyRBE) and (B) an intensity-modulated radiation therapy treatment plan (mean heart dose, 12.7 Gy). The clinical target volume 1 is outlined in yellow. Abbreviation: PBS, pencil-beam scanning.

The median mean cardiac dose was 2.2 (range, 0.1-4.96) Gy. Patients treated with protons alone had the lowest mean cardiac doses (median 0.7 versus 3, respectively). Median left lung V5 and V20 were 34.9% (range, 19.9%-55%) and 19.2% (range, 4.9%-36.5%), respectively. Representative dosimetry is shown in **Figure 4**.

### Disease Control

No patient experienced a local recurrence. Three of 4 patients who received preoperative HART achieved a pathologic complete response. The other patient treated preoperatively had an excellent treatment response with 0.7 cm of residual disease, treatment effect present, and 0 mitosis per 10 high-powered fields. Surgical margins were negative in all preoperative HART cases. A PET/CT scan demonstrated a complete metabolic response 5 months after RT in the patient treated with definitive HART. She was still recurrence-free 16 months from the start of RT. Two patients developed metastatic disease, one with lung metastases and the other with diffuse bone metastases, and died of their disease 19 months and 21 months after RAAS diagnosis.

### Toxicity

**Figure 5.**
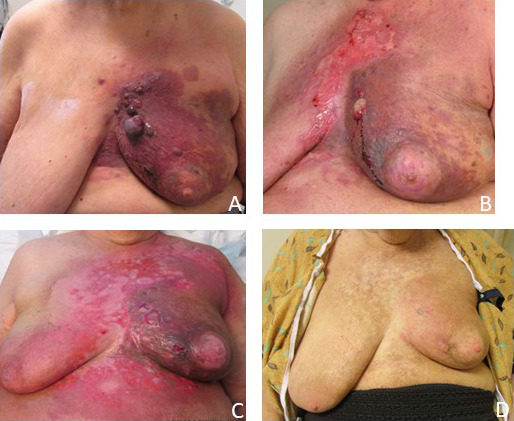
(A) Clinical presentation at the start of radiation therapy. (B) Partial tumor response and associated radiation dermatitis present at the completion of radiation therapy. (C) Radiation dermatitis 13 days after radiation therapy. (D) Clinical appearance 11 months after radiation therapy with no evidence of progressive disease.

There were no cases of ≥ grade 4 acute toxicity. Three patients developed grade 3 dermatitis. The peak intensity of dermatitis occurred in the second or third week after completion of HART (**Figure 5**). Two patients experienced grade 3 fatigue while on treatment. One patient with chronic kidney disease required intravenous fluids owing to a mild increase in potassium and creatinine due to decreased fluid intake secondary to fatigue. No other patients developed electrolyte abnormalities. One patient with longstanding hypertension, diabetes, chronic obstructive pulmonary disease, and stage III kidney dysfunction developed asymptomatic grade 2 bilateral pleural effusions 5 months after completing HART; cytology demonstrated benign findings, and she underwent a limited thoracocentesis with resolution of symptoms. She was diagnosed with congestive heart failure and her symptoms resolved with medical management. Patients 1 and 6, who received preoperative HART, developed grade 2 wound dehiscence after mastectomy associated with slow wound healing. Patient 3, who also received preoperative HART, developed a grade 3 wound infection following mastectomy and was treated successfully with antibiotics. No patients had experienced rib fractures or chronic chest wall pain. No patients experienced grade 3 late toxicity. Two patients developed grade 2 superficial soft-tissue fibrosis. Patient 4, treated with postmastectomy HART, experienced grade 2 acute dermatitis manifested by moist desquamation in the axilla, which persisted as a chronic grade 2 wound in the setting of chemotherapy for metastatic progression.

## Discussion

The management of RAAS is challenging—the disease is aggressive, and patients are often elderly. An oncologic resection alone can fail to provide adequate local control [24]. Even with uninvolved margins, recurrences may occur owing to the infiltrative nature of RAAS [3, 22, 25, 27, 28, 36, 37]. The 2 patients in this series who underwent upfront mastectomy (patients 4 and 6) exemplify this problem; despite uninvolved margins, the disease rapidly recurred within 9 weeks after mastectomy.

### Imaging

Consistent with prior reports, skin changes comprised the main clinical findings at presentation in our study. Four of the 6 patients had no evidence of malignancy on screening mammogram within 12 months of their diagnosis of RAAS. Patient 5, who had dermal sclerosis, did have a mammogram demonstrating an interval worsening of skin thickening, which prompted workup that led to her RAAS diagnosis. Our findings are concordant with prior literature and highlight that mammography often demonstrates normal findings or only nonspecific findings, such as skin thickening, and is unreliable for surveillance/detection of RAAS [8, 25, 38–41]. We found PET/CT particularly useful; extensive skin thickening (>5 cm) with mild to moderate uptake was evident in all cases (median standard uptake value, 2.8; range, 1.9-12.5); coregistration of PET/CT images with CT simulation images facilitated target delineation in conjunction with clinical findings. In addition, PET imaging confirmed the absence of overt metastatic disease.

While imaging is helpful to characterize disease extent, the diagnosis is clinical. Despite its rarity, clinicians should remain vigilant to the possibility of RAAS in patients previously treated with RT and maintain a high index of suspicion for any cutaneous abnormalities that arise in a previously irradiated breast; there should be a low threshold for further investigations [20].

### Disease Control with HART

Radiation therapy is highly effective, but its use has been limited owing to the large volumes treated and the expected cumulative doses of RT, which can exceed 110 Gy. Despite the inherent risks related to reirradiation, long-term data have demonstrated that RT can be delivered efficaciously and safely with HART [3]. Smith et al [3] evaluated the use of HART for 14 patients treated at the University of Florida and demonstrated 5- and 10-year cause-specific survival rates of 79% and 71%, respectively. These findings suggest that long-term control and, likely, cure, of RAAS is possible by using a combination of HART with or without surgery.

Surgery alone without adjuvant therapy is associated with poor local control. A recent study evaluated radical chest wall resection. Li et al [23] reported the outcomes of 76 women with RAAS, half of whom were treated with an aggressive surgical approach similar to that described by Donovan et al [42], but with the omission of adjuvant RT, and the other half treated with a more traditional surgical approach. With a median follow-up of 27 months, the 5-year local recurrence rate was 23% and 76% in the groups with radical surgery and more conservative surgery, respectively. The median time to local recurrence was 8 months. Interestingly, multifocality was demonstrated more often in radical-resection specimens (80%) than conservative-resection specimens (56%), raising the possibility of missed foci of tumor by conservative resection. Moreover, the authors questioned the reliability of negative margin assessment owing to the multifocal nature of RAAS. An essential aspect of the study by Donovan et al [42] was the use of a soft-tissue sarcoma approach to surgery rather than routine mastectomy. The standard surgery in this study—radical chest wall resection with an en bloc mastectomy—aimed not only for an oncologic resection but also for resection of as much originally irradiated tissue as possible. The authors based this practice on data showing that local recurrence primarily occurs in previously irradiated tissue [8, 42–44]. The pectoralis fascia was resected in all cases. In agreement with Smith et al [3], wound closure involved autologous tissue transfer in the form of local/regional flaps after resection. A retrospective study of 50 patients with RAAS from the Finnish Cancer Registry reported a 5-year local recurrence-free survival rate of 62% with surgical resection, the mainstay of therapy, and a median pathologic margin of 2 (range, 0-6.0) cm [41]. Only 1 patient received adjuvant RT and 5 received adjuvant chemotherapy. Three patients had inoperable disease and died within 2 months. Of the 47 patients with operable disease, 32% had resection of a pectoral muscle, 70% had resection of the pectoralis fascia, and 21% required an additional operation owing to close margins. Surgical reconstruction was performed in 51%, most commonly by using a pedicled latissimus dorsi flap with or without skin grafts. Local recurrence developed in 21 of the 47 patients with operable disease, 19 of whom underwent additional surgery, with 7 developing distant disease and 5 with a second local recurrence, 1 of whom was alive at last follow-up. The 5-year distant recurrence-free survival rate was 75% and the overall survival rate was 74%.

Highly aggressive tumors such as RAAS can exhibit rapid repopulation during RT that can result in tumor progression beyond the initial target volume [27, 45]. Owing to extensive tissue infiltration [10, 23, 46], inadequate treatment margins can cause a geographic miss of subclinical disease. To overcome the inherently aggressive biology of RAAS, HART differs from conventional RT in 3 ways:

1. Hyperfractionation: smaller fraction sizes can reduce late toxicity [47]. Three fractions are delivered daily on weekdays (TID). To allow time for repair of sublethal normal tissue injury, patients treated with the TID regimen receive 1 GyRBE per fraction with a minimal 4-hour interfraction interval.
2. Acceleration: HART delivers the total dose in a shorter time frame than conventional fractionation to overcome tumor repopulation. The total daily dose is 3 GyRBE, compared to 1.8 GyRBE to 2 GyRBE in conventional fractionation.
3. Large elective target volumes: Treatment margins are generous owing to the infiltrative nature of RAAS that results in a high probability of occult disease within clinically and radiologically normal-appearing tissues.

HART has been successfully used by other institutions in the management of RAAS [48–50]. A recent Canadian study involving 9 patients reported a crude local recurrence rate of just 11.1%, with a median follow-up of just 19 (range, 3-41) months and prescription doses of 45 to 60 Gy, determined by margin status [42]. Interestingly, the results challenge the notion that HART is best delivered preoperatively; HART was delivered exclusively in the adjuvant setting with favorable outcomes and without incidence of severe toxicity. The authors adopted an adjuvant approach owing to concerns about wound healing with preoperative RT, which is associated with higher wound complication rates with surgery [51].

The role of systemic therapy for RAAS in the setting of HART remains an area of investigation. RAAS appears to be at least moderately responsive to chemotherapy, and preliminary data suggest that adjuvant chemotherapy may improve survival outcomes [19, 29, 52, 53]. Data also have demonstrated the potential effectiveness of tyrosine kinase inhibitors and immunotherapy [54–61]. Unfortunately, attempts to include systemic therapy may be hampered by most patients' advanced age, performance status, and the convalescence period needed after an intensive regimen of HART and surgery.

While continued follow-up is necessary to surveil later local recurrence and metastatic progression, HART with proton therapy shows promising early results, consistent with early disease control rates from prior nonproton series [3]. All patients in our series had clinical and radiologic evidence of extensive disease, most achieved a pathologic complete response, and none developed a local recurrence (5 of 6 patients had follow-up exceeding 16 months). This series adds to a case report published on proton therapy for breast angiosarcoma in which patients were treated postoperatively with conventional fractionation to a median dose of 60 Gy [62]. Similar to our series, favorable dosimetry was achieved with proton therapy.

### Toxicity with Proton Therapy

Acute skin toxicity was often deceptively mild during treatment, with the peak severity occurring 2 to 3 weeks after RT. Hence, we recommend weekly surveillance for at least 3 weeks after completing RT. Two patients experienced slow wound healing. One did not have reconstruction with autologous tissue transfer, which can aid in wound healing [3]. One elderly patient with multiple baseline risk factors developed congestive heart failure <6 months after RT, which, considering the time to diagnosis, may have been present but undiagnosed before RT. This patient had a large irradiated field and a mean heart dose of nearly 5 Gy. We recorded no grade 3 late toxicity in our study, with limited follow-up. No patients have developed pneumonitis or rib fracture. Smith et al [3] reported a higher risk of severe toxicity at the matchline between electron fields due to the inherent dose heterogeneity associated with field junctions, despite feathering techniques. In their study of 14 patients, 2 developed hyperpigmentation, subcutaneous fibrosis, and pruritus at the matchlines, requiring the use of selenium. In our series that included feathered double-scattered proton, electron, and photon-electron matchlines, we did not observe increased skin toxicity at the junctions.

We recognize that the small sample size and limited follow-up of this study preclude us from comprehensively assessing the efficacy and toxicity of HART delivered with proton therapy for RAAS. Additionally, proton therapy was not the sole modality for 3 of our patients, as evidenced by the higher mean cardiac doses among patients who received some photon therapy. Nonetheless, our data demonstrate the feasibility of using protons to deliver HART and helps inform the management of RAAS, for which data are scarce.

## Conclusion

RAAS is a potentially curable condition, and HART combined with surgery has yielded promising results. In this setting of large-volume reirradiation with long-term survival, minimizing acute and late toxicity is increasingly relevant. HART delivered with proton therapy has a high treatment response rate and appears effective in local control of RAAS, but systemic disease remains an important cause of mortality. Proton therapy should be considered in the treatment of RAAS to maximize normal-tissue sparing.
