# Supplementary material for: Hyperfractionated-Accelerated Reirradiation with Proton Therapy for Radiation-Associated Breast Angiosarcoma
Source: Int J Part Ther. 2022 Jan 18;8(4):55–67. doi: 10.14338/IJPT-21-00031.1 (PMC9009453; doi:10.14338/IJPT-21-00031.1)
Supplement: Supplementary file 1 [file ijpt-08-04-09_s01.pdf]

Supplementary Figures 1 through 6: Clinical Presentation of Each Patient (N=6)

Figure S1:

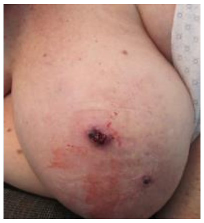

Patient 1

Figure S2:

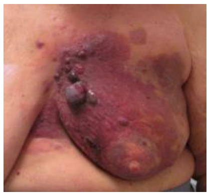

Patient 2

Figure S3:

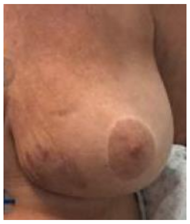

Patient 3

Figure S4:

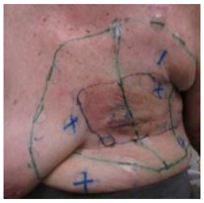

Patient 4

Figure S5:

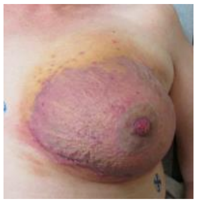

Patient 5

Figure S6:

Initial:

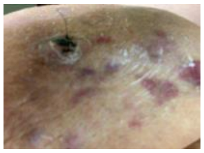

Recurrent:

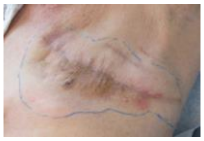

Patient 6
