# Supplementary material for: Hyperfractionated-Accelerated Reirradiation with Proton Therapy for Radiation-Associated Breast Angiosarcoma
Source: Int J Part Ther. 2022 Jan 18;8(4):55–67. doi: 10.14338/IJPT-21-00031.1 (PMC9009453; doi:10.14338/IJPT-21-00031.1)
Supplement: Supplementary file 2 [file ijpt-08-04-09_s02.pdf]

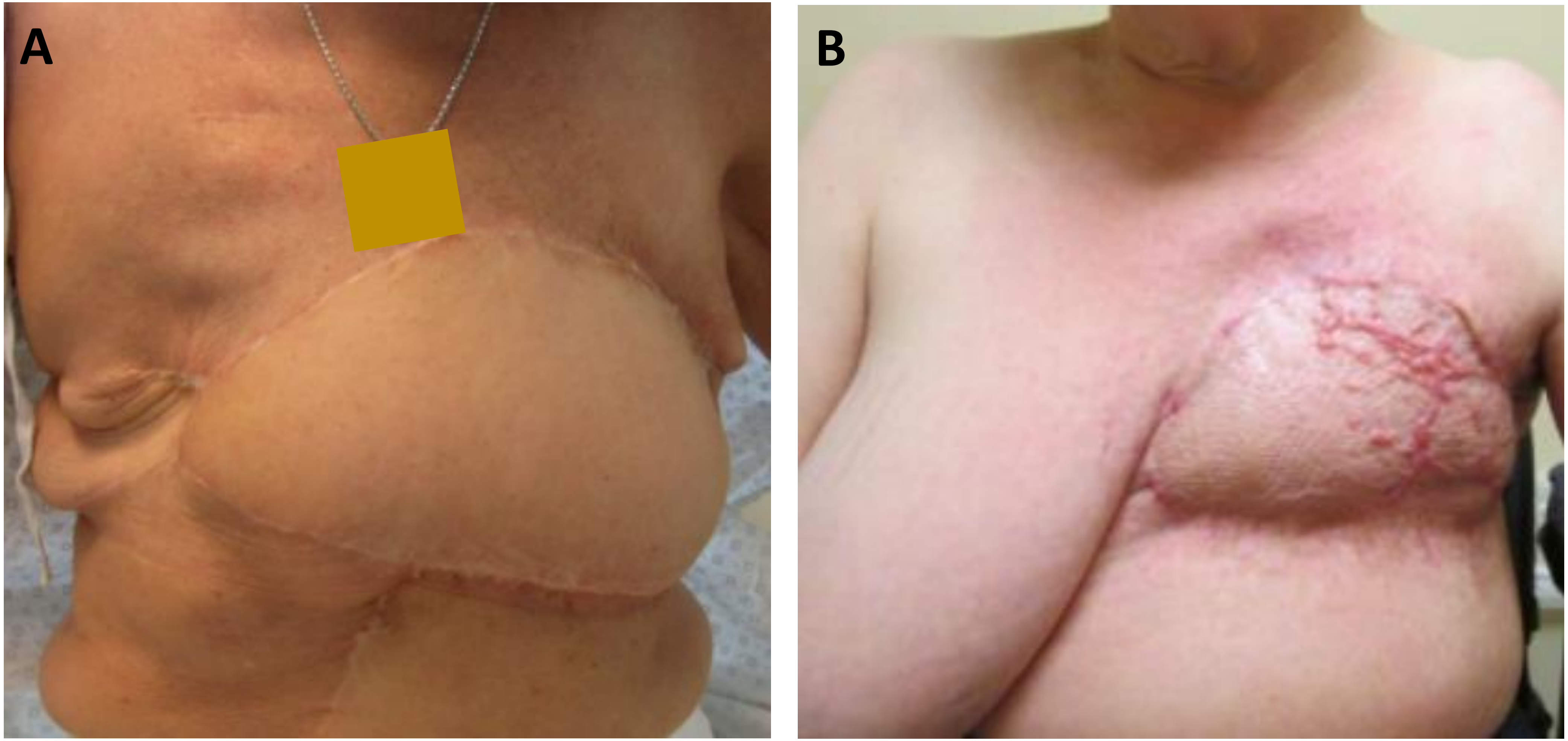

Supplementary Figure 7. Post-treatment appearance of the affected breast. (A) Patient 3 at 34 months after preoperative HART. (B) Patient 5 at 6 months after completion of preoperative HART
